# A Common Path to Innate Immunity to HIV-1 Induced by Toll-Like Receptor Ligands in Primary Human Macrophages

**DOI:** 10.1371/journal.pone.0024193

**Published:** 2011-08-31

**Authors:** Xingyu Wang, Wei Chao, Manisha Saini, Mary Jane Potash

**Affiliations:** Molecular Virology Division, St. Luke's-Roosevelt Hospital Center, Columbia University Medical Center, New York, New York, United States of America; National Institute of Allergy and Infectious Diseases - Rocky Mountain Laboratories, United States of America

## Abstract

Toll-like receptors (TLR) represent the best characterized receptor family transducing innate immune responses, the first line of defense against microbial invaders. This study was designed to investigate whether responses through TLR inhibit HIV-1 replication in its primary target cells. Primary human macrophages and lymphocytes from several different donors and HIV-1 infection in tissue culture were used exclusively in this work. We report that ligands of three different TLR: LPS, R848, and double stranded RNA, induce a common antiviral response in macrophages as assayed by measurement of HIV-1 p24 protein, *gag* DNA, and entry into cells. HIV-1 infection is arrested after efficient entry but prior to reverse transcription. TLR-ligand activated cells secrete antiviral factors that induce a similar restriction. HIV-1 infection of lymphocytes is not affected by exposure to TLR ligands or to antiviral factors secreted by activated macrophages. TBK1, but neither NF-κB nor JAK-STAT activity, is required in macrophages to mount this antiviral response; the combination of p38 MAPK and JNK are partially required for induction of antiviral activity. Based on transcriptional induction and inhibition, the TLR-linked antiviral activity is different from APOBEC3 A or G, interferon-β, NAMPT, or p21^Cip1^. The cell-type specificity, site of action, and requirement for signaling intermediates suggest that the TLR-linked antiviral activity is novel.

## Introduction

Macrophages perform dual roles during HIV-1 infection [1]. Like CD4-bearing T lymphocytes, they are a prominent target for virus replication. As major effectors of innate immunity, they have the potential to mount antiviral responses within hours of systemic infection. The salience of such immediate reaction to HIV-1 infection has been demonstrated in the intense cytokine cascade detected in the plasma of HIV-1 infected people that precedes the peak of viremia seen early after virus transmission [2].

Innate immune responses can be initiated by triggering pattern recognition receptors (PRR) that bind classes of molecules expressed by pathogens [3], [4]. The first family of PRR defined in mammals is the Toll-like receptor (TLR) group that comprises both plasma membrane and intracellular receptors for bacterial or viral lipids, proteins, or nucleic acids. TLR expression is cell type specific with dendritic cells and macrophages expressing all TLR. TLR ligation activates distinct signal transduction pathways resulting in transcription of many effector molecules including TNF-α, IL-6, and type I interferons [4].

With diverse TLR types and diverse ligands, it is not surprising that innate immune responses through TLR have been observed to have several different effects upon HIV-1 replication. More than 20 years ago it was recognized that the commonly used macrophage activator and TLR4 ligand LPS reduces HIV-1 replication in primary human macrophages [5]. The basis of this inhibition has been attributed to impaired viral entry by down-modulation of CCR5 [6] or by an unknown soluble factor inhibiting X4-tropic virus entry [7]. Prothymosin-α, also by ligation of TLR4, was shown to induce type I interferon production by macrophages and inhibit HIV-1 replication after viral DNA integration [8]. Mycobacterium tuberculosis, which contains ligands for TLR2 and TLR4, has been shown to induce a post-entry, pre-reverse transcription block in HIV-1 replication in macrophages [9], although earlier studies had shown that mycobacterium infection of macrophages enhanced HIV-1 replication [10]. One study investigating several TLR responses found that the TLR5 ligand, flagellin, enhanced both R5 and X4 HIV-1 replication while a TLR9 ligand, M362, inhibited replication by both viruses in lymphoid tissue blocks [11]. Ligation of TLR3 induced multiple antiviral activities in primary human macrophages and blocked HIV-1 replication [12].

We have a long-standing interest in HIV-1 replication in macrophages and its control. The present study was designed to determine how innate immune responses affect HIV-1 replication by investigating common effects of different TLR ligands upon HIV-1 infection of monocyte-derived macrophages (MDM). We found that ligation of TLR3, 4, or 7/8 on MDM blocked R5 HIV-1 infection of MDM but not of peripheral blood lymphocytes. After TLR activation, MDM secreted a soluble factor that inhibited HIV-1 infection of untreated MDM. Infection was arrested after virus entry into MDM but before reverse transcription. Using pharmacological inhibitors we found that TLR activation to this antiviral state did not require NFκB, JAK, JNK, or but did require TBK1. The antiviral state triggered by TLR activation could be distinguished from the induction of Type I interferon, ABOBEC3G, p21^Cip1^, and NAMPT. Taken together our results indicate that TLR activation of human MDM induces the production of a potentially novel antiviral activity blocking HIV-1 infection following viral internalization.

## Results

**Figure 1:**
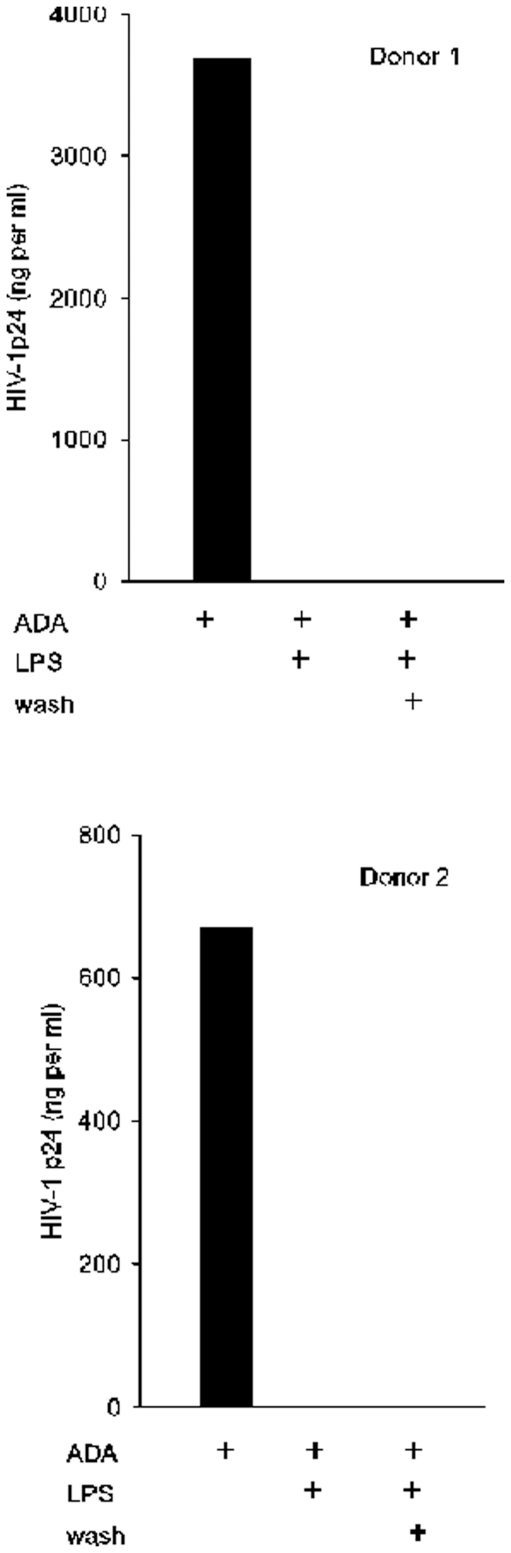
LPS induces an anti-HIV-1 response in MDM. MDM from two different donors were treated with vehicle or LPS prior to and during infection with ADA. LPS was washed out with virus (wash) or was replaced for one week culture. Cell supernatants were then harvested for measurement of HIV-1 p24. N (number of experiments with cells from different donors) >4.

**Figure 2:**
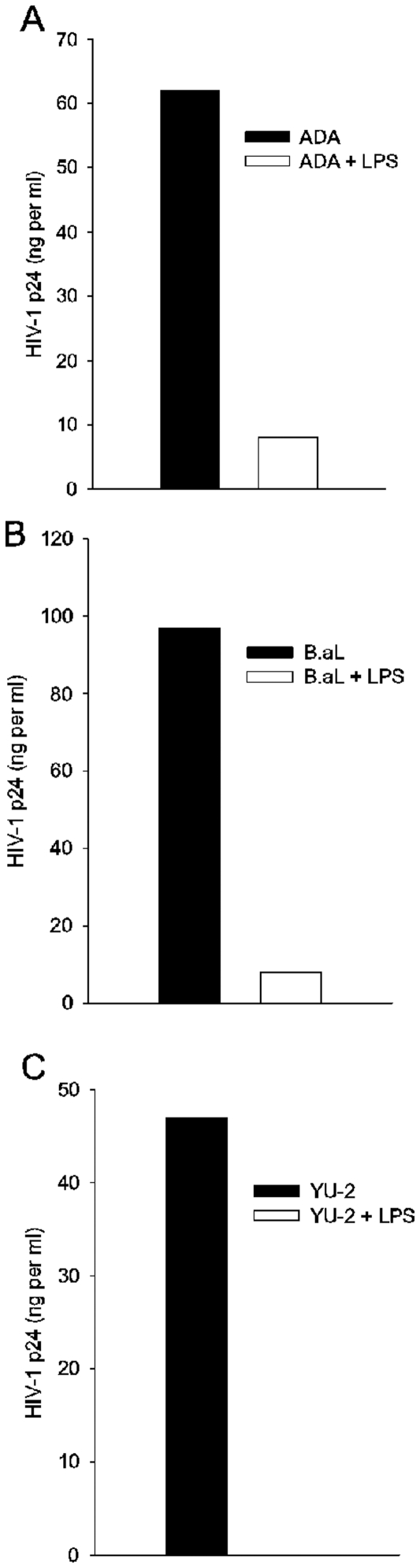
LPS activated MDM resist infection by three different strains of HIV-1. MDM were treated with LPS or vehicle and infected with the virus indicated, LPS was washed out with virus. After one week of culture, cell supernatants were harvested for measurement of HIV-1 p24. N = 2.

For an overview of the effects of TLR-ligation on HIV-1 infection of MDM, cells from two different donors were treated with LPS, a TLR4 ligand, at the time of infection by ADA and either washed out with virus or replaced after washing and maintained during one week culture. HIV-1 replication was monitored by measurement of extracellular p24 one week after infection during the exponential increase in p24 production we timed during studies of MDM infection kinetics [13] (Fig. 1). With both transient and maintained exposure, LPS blocked ADA replication in macrophages more than 100-fold. To determine whether this anti-HIV-1 response restricts only the HIV-1 strain ADA, we tested the sensitivity of other R5 HIV-1 strains to inhibition by transient exposure to LPS. MDM were treated with LPS and infected either with ADA, B.aL, or YU-2 and infection was monitored by p24 expression (Fig. 2). MDM susceptibility to each virus was greatly inhibited by exposure to LPS.

**Figure 3:**
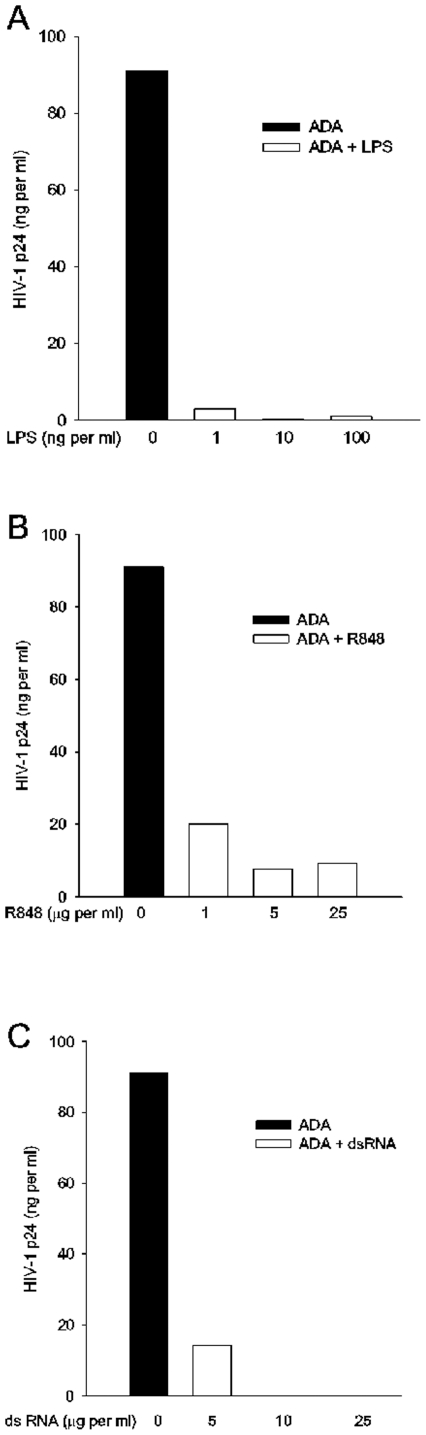
Three different TLR ligands induce an anti-HIV-1 response in MDM. MDM were treated with either vehicle, LPS, R848, or dsRNA at the doses indicated. Cells were treated and infected as described in Fig. 1 with ligands washed out with virus. N = 3.

To determine whether this antiviral effect was common to different TLR responses, the experiment was repeated with MDM that were treated in dose response either with LPS; R848, a synthetic TLR7/8 ligand; or double stranded RNA, a TLR 3 ligand; during ADA infection, each TLR ligand was washed out with virus for transient exposure. Virus replication was monitored by measurement of extracellular p24 four days after infection (Fig. 3). The innate immune response through TLR3, 4, or 7/8 each controlled HIV-1 infection of primary human macrophages. In contrast, neither of the macrophage activators, TNF-α nor supernatants of primary human astrocytes, significantly affected HIV-1 replication in MDM (not shown) [14], [15].

**Figure 4:**
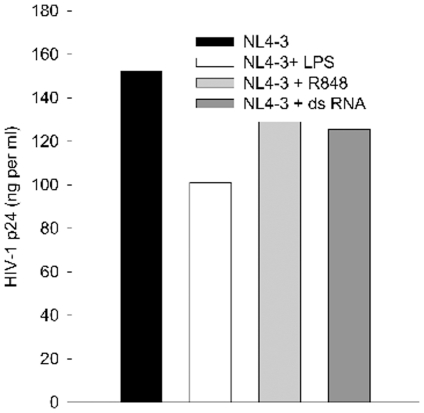
PBL fail to respond to TLR by an anti-HIV-1 response. Mitogen activated PBL were treated with TLR ligands as described in Materials and Methods prior to and during exposure to NL4-3. Virus and TLR ligands were washed out, cells returned to culture for one week prior to collection of supernatants for measurement of HIV-1 p24. N = 2.

A recent study shows that HIV-1 infection of lymphoid tissue is affected differently by different TLR ligands [11], so we investigated whether HIV-1 infection of purified human peripheral blood lymphocytes (PBL) is affected by exposure to ligands of TLR3, 4, or 7/8. Mitogen activated PBL were treated with dsRNA, LPS, or R848 and then infected with X4 HIV-1/NL4-3 and infection was monitored by measurement of extracellular p24 after one week (Fig. 4). In contrast to MDM infection, PBL infection was only minimally affected by any TLR ligand suggesting that the response is cell-type specific.

**Figure 5:**
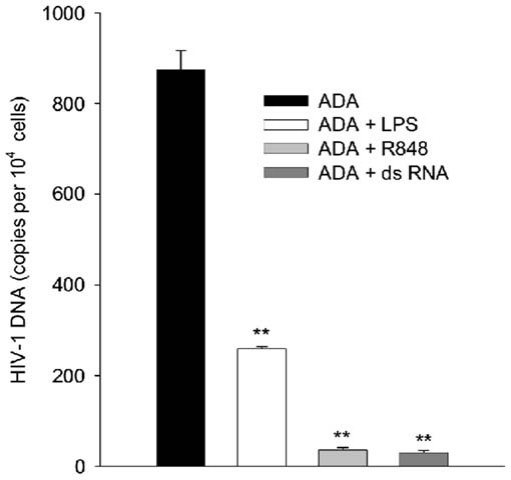
TLR ligand induced anti-HIV-1 response blocks infection prior to reverse transcription. MDM were treated with TLR ligands as described in Materials and Methods prior to and during exposure to ADA. Virus and TLR ligands were washed out, cells returned to culture for 24 h prior to collection of cells for measurement of HIV-1 *gag* DNA by real-time PCR; means and standard deviations are plotted, ** p<0.005 in Student's *t* test of virus burdens between vehicle and TLR treated cells. N = 4.

Endogenous antiviral activities act at several phases of the HIV-1 life cycle [16] so we investigated at what stage of HIV-1 replication the TLR response of MDM exerts its effects. Cells were treated either with LPS, R848, or dsRNA; infected with ADA and after 24 h, during the first round of reverse transcription in infected MDM [13], [17], viral *gag* DNA was measured by real-time PCR; standardizing DNA by amplification of β globin (Fig. 5). As observed with measurement of infection by p24 production, MDM responded to different TLR ligands in the same way, here by arresting ADA infection prior to viral DNA synthesis. Since HIV-1 infection is arrested before reverse transcription potential sites of TLR ligand induced inhibition later in the virus life cycle are rendered moot.

**Figure 6:**
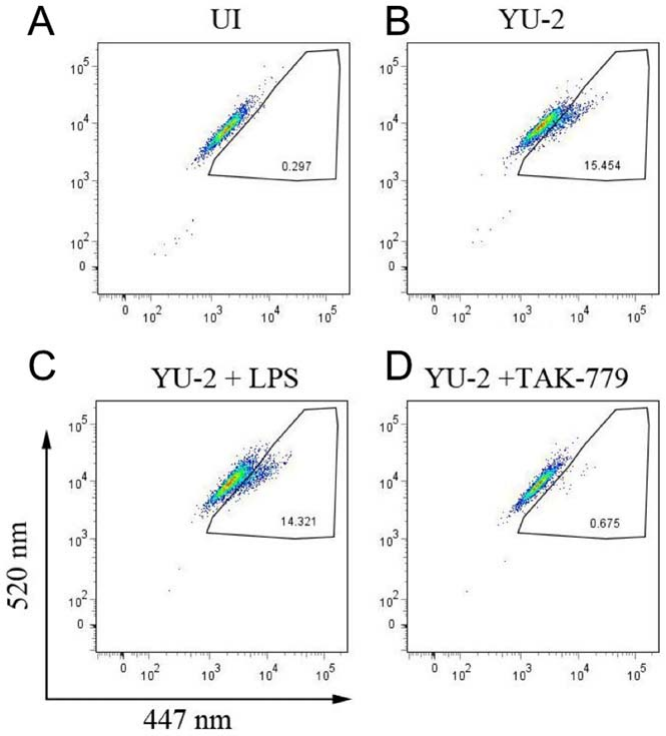
LPS activated MDM permit efficient HIV-1 entry. MDM were treated with vehicle (B) LPS (C), or TAK-779 (D), infected with YU-2-Blam-Vpr, and subjected to flow cytometry as described in Materials and Methods. Uninfected cells (A) serve as negative controls. N = 6.

To further define the site of infection arrest, we employed an assay of HIV-1-cell fusion in which Vpr-β lactamase (Vpr-BLaM) is encapsidated in HIV-1 virions and virus entry permits cleavage of a BLaM substrate loaded into cell cytoplasm, cleavage is scored by a fluorescence shift from green to blue [18] (Fig. 6). MDM were treated either with LPS or with TAK779, a CCR5 antagonist, then infected with YU-2 containing Vpr-BLaM, and then assayed for fusion (Fig. 6), or were cultured in parallel to measure p24 levels. MDM allowed efficient entry of YU-2 that was completely sensitive to neutralization by TAK779. In sharp contrast to their block on viral DNA synthesis (Fig. 5), LPS-treated MDM were highly susceptible to HIV-1 entry (Fig. 6) ruling out viral entry inhibitors potentially induced by LPS in the antiviral effect observed. MDM treated transiently with LPS restricted YU-2 infection with control cells producing 47 ng p24 per ml and LPS-treated cells producing less than 2 ng p24 per ml.. Results shown in Figures 5 and 6 indicate that LPS treated MDM arrest HIV-1 infection after efficient binding, fusion, and entry but prior to reverse transcription.

**Figure 7:**
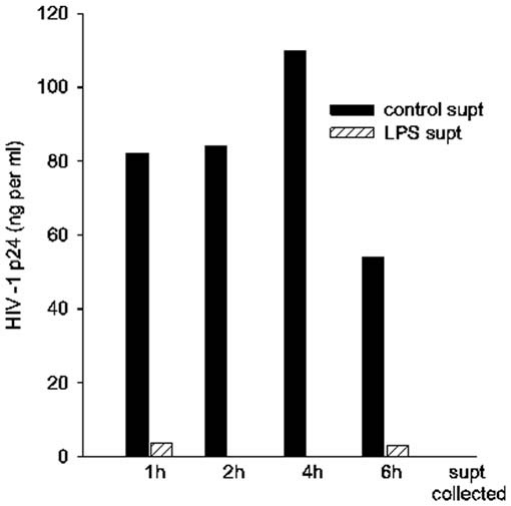
LPS activated MDM secrete anti-HIV-1 factor (s). MDM were treated with LPS for 10 min or left untreated, washed, and then supernatants harvested at the times shown. Supernatants were then applied to MDM prior to ADA infection. Infected MDM were cultured four days prior to collection of supernatants for measurement of p24. N>3.

**Figure 8:**
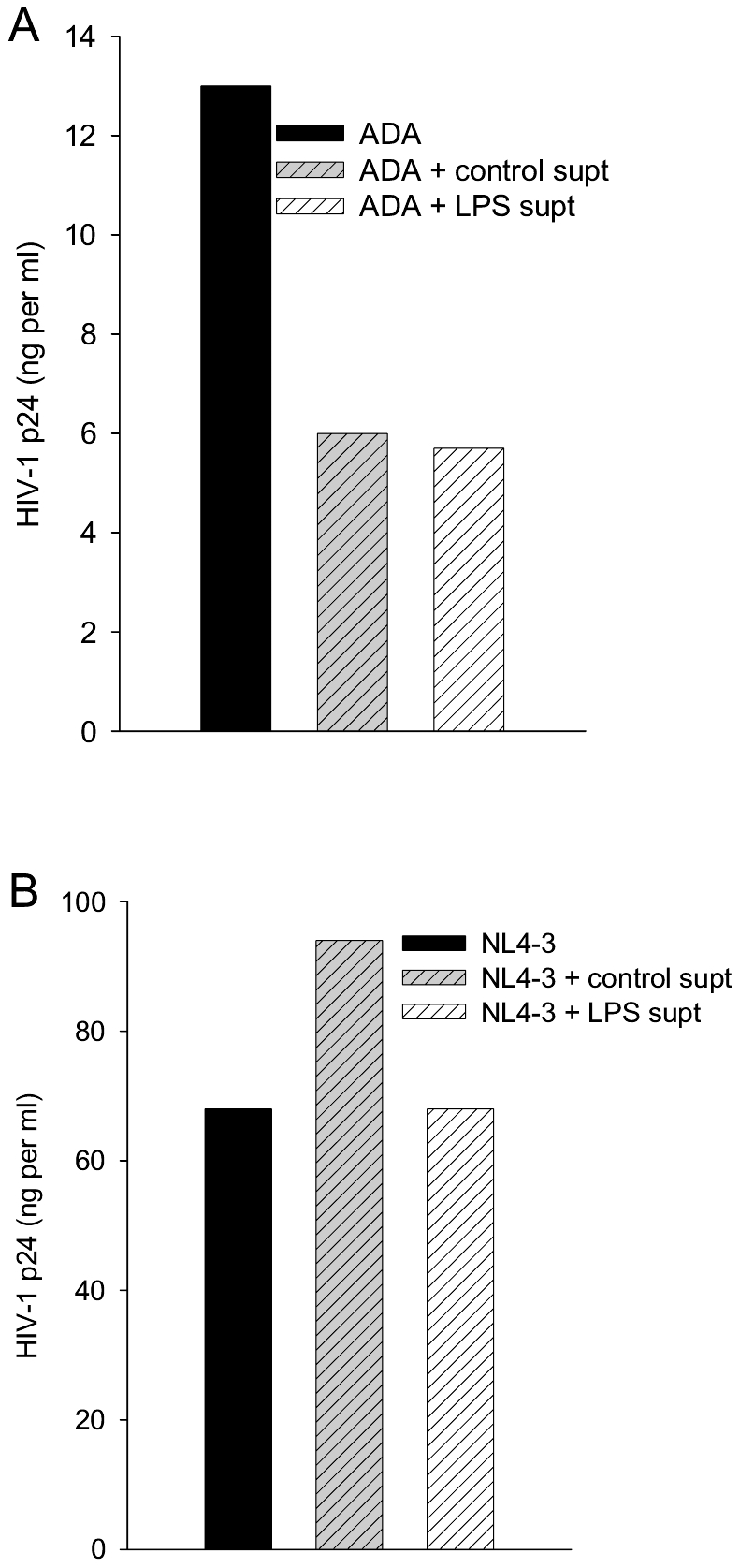
HIV-1 infection of PBL is resistant to LPS-induced antiviral factor. Supernatants of MDM activated by LPS were harvested after 4 h and applied to PBL prior to infection by ADA (A) or NL4-3 (B). Infected PBL were cultured for one week prior to collection of supernatants for measurement of p24. N = 2.

TLR-stimulation activates secretion of several physiologically active factors, so we tested the supernatants of MDM treated with LPS for various times to determine whether they contain an antiviral activity. MDM were treated with LPS or left untreated, washed extensively, and then supernatants were collected over several hours. For infection MDM were treated with supernatants during exposure to ADA and extracellular p24 was measured four days after infection. LPS-treated MDM secrete an anti-HIV-1 factor(s) that largely prevents infection of MDM; its activity is present within one hour of LPS stimulation and persists for several hours thereafter (Fig. 7). To begin to characterize the TLR-induced antiviral factors, we tested their ability to block HIV-1 infection of PBL that themselves do not mount antiviral responses by exposure to TLR ligands (Fig. 4). Supernatants of control MDM or LPS-activated MDM known to block HIV-1 infection of MDM were tested for their effects upon HIV-1 infection of PBL (Fig. 8). It is striking that PBL retained full susceptibility to X4 NL4-3. The two-fold decrease in PBL susceptibility to R5 ADA after exposure to supernatants of both control and activated MDM may indicate the presence of similar levels of ß–chemokines that inhibit R5 HIV-1 entry [19].

**Figure 9:**
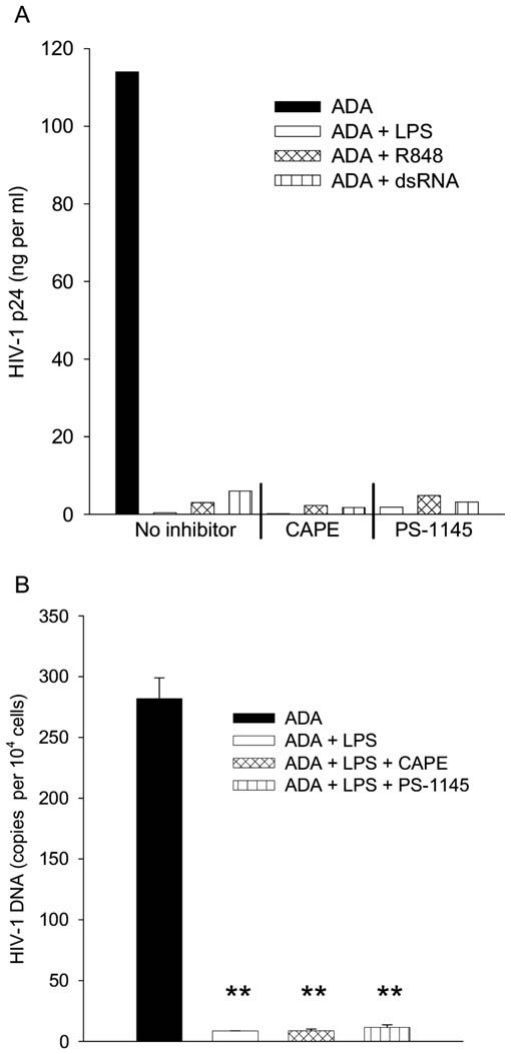
NF-κB is dispensable for the anti-HIV-1 response induced by three different TLR ligands. (A) MDM were activated and infected by ADA as described in Fig 3 in the presence of CAPE, PS-1145, or vehicle and were cultured four days prior to collection of supernatants for measurement of p24. N = 2. (B). MDM were treated with the inhibitors shown and LPS and then supernatants were harvested; supernatants were then applied to MDM during ADA infection as described in Materials and Methods; infected cells were harvested after 24 h for measurement of HIV-1 DNA, means and standard deviations are plotted. N = 3. ** p<0.005 in Student's *t* test of virus burdens between vehicle and LPS treated cells.

TLR responses can utilize multiple signaling pathways [20]. To identify key elements leading to the production of inhibitors of HIV -1 infection, we activated MDM with TLR ligands in the presence of inhibitors of various signaling intermediates and assayed their susceptibility to HIV-1 infection. Given its relevance to TLR responses, the role of NF-κB activation in antiviral factor induction was first tested, using CAPE that interferes with the binding of NF-κB to DNA and PS-1145, which inhibits phosphorylation of I-κB. Pilot electrophoretic mobility shift studies confirmed that CAPE and PS-1145 inhibited LPS-induced activation of NF-κB in MDM under conditions used here (not shown). To test the role of NF-κB in LPS-induced antiviral activity, MDM were pretreated with inhibitors for 1 h, activated with different TLR ligands in the presence of inhibitors, infected by ADA, and then cultured for four days, virus replication was monitored by p24 production (Fig. 9A). Neither inhibitor of NF-κB activation had an effect upon the complete HIV-1 inhibition induced by LPS, R848, or dsRNA. However both CAPE and PS-1145 themselves inhibited ADA replication two to three fold and the mechanism of this inhibition is under investigation. Testing supernatants of similarly activated MDM for their effects upon ADA replication prior to reverse transcription confirmed that induction of an antiviral state was not dependent upon NF-κB (Fig. 9B).

**Figure 10:**
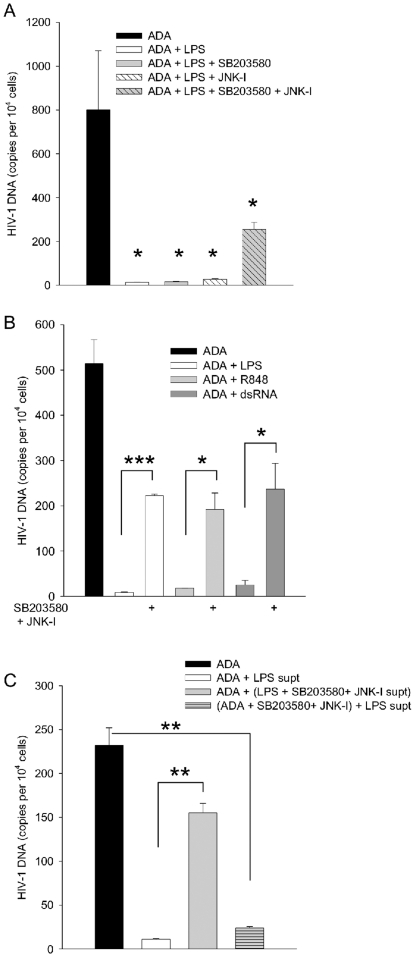
The combined effects of p38 and JNK MAPK are partially required for the anti-HIV-1 response induced by three different TLR ligands. Panel A. MDM were activated by LPS and infected by ADA in the presence of SB203580, JNK-I, both inhibitors, or vehicle. N = 3. Panel B. MDM were activated by LPS, R848, or dsRNA and infected by ADA in the presence of SB203580 and JNK-I or vehicle. N = 2. Panel C. MDM were activated by LPS in the presence of vehicle or SB203580 and JNK-I (LPS + SB203580+ JNK-I supt, grey bar). Supernatants were collected and applied to fresh MDM that were infected by ADA in the presence of vehicle or SB203580 and JNK-I (ADA+ SB203580+JNK-I, grey striped bar). N = 3. All infected cells were harvested after 24 h for measurement of HIV-1 DNA, means and standard deviations are plotted. * p<0.05, ** p<0.005, *** p <0.0005 in Student's *t* test of virus burdens infected cell systems indicated.

Using the same logic to identify intermediates in control of gene expression leading to an antiviral state, we investigated the requirements for p38 MAPK and JNK, kinases required for the TLR induction of expression of some inflammatory cytokines for effects upon the HIV-1 resistance in MDM. We scored HIV-1 replication and inhibition by the measurement of viral DNA (Fig. 10A). Tested alone, neither the JNK MAPK inhibitor (JNK-I) nor the p38 MAPK inhibitor, SB203580, affected the LPS antiviral response, however when the inhibitors were tested together there was a partial relief in the LPS block to HIV-1 infection. When tested in the absence of TLR ligands, we found no effect of the JNK-I and SB203580 upon ADA infection (not shown). To confirm the requirement for these kinases in TLR responses, we tested the effects of R848 and dsRNA as well as LPS for effects upon HIV-1 replication in the presence of SB203580 and the JNK-I (Fig. 10B). Anti-HIV-1 responses to any of the three TLR ligands were partially reversed by blocking the combination of these kinase cascades. Similarly, supernatants of MDM activated by LPS in the presence of SB203580 and the JNK-I contain less antiviral activity (Fig. 10C). This observation is consistent with a requirement for p38 MAPK and JNK in the response to LPS producing an antiviral factor or in the action of the antiviral factor in blocking HIV-1 replication. To distinguish between these possibilities, we separated LPS activation of MDM from test of antiviral activity during HIV-1 infection. MDM were activated with vehicle or LPS in the presence or absence of SB203580 and the JNK-I and their supernatants were harvested to assay antiviral activity. Antiviral activity was tested during ADA infection of MDM, conducted in the presence or absence of SB203580 and the JNK inhibitor (Fig. 10C). The combination of SB203580 and JNK-I reduced the level of antiviral activity in supernatant of LPS treated cells. However, that the action of the antiviral factors in supernatants of LPS-activated cells is independent of both p38 MAPK and JNK, since MDM treated with LPS supernatants were resistant to ADA infection, despite being infected and cultured in the presence of the kinase inhibitors (Fig. 10C).

**Figure 11:**
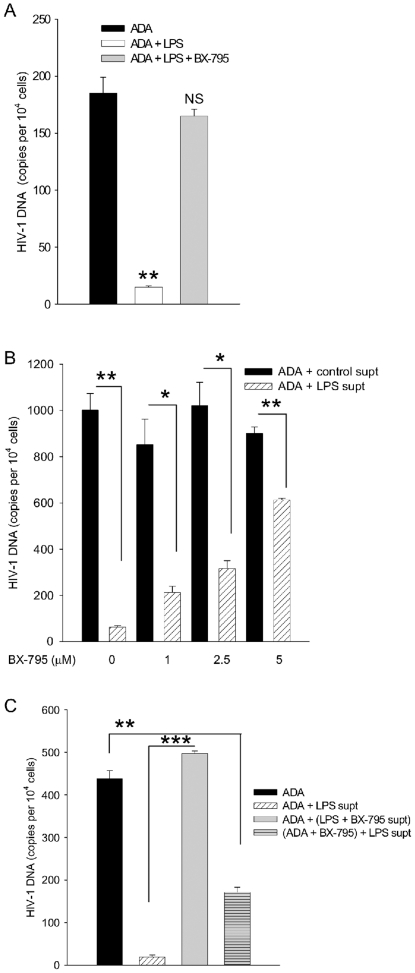
TBK1 is required for the anti-HIV-1 response induced by LPS. (A) MDM were activated by LPS and infected by ADA in the presence of BX-795 or vehicle. N = 3. (B) MDM were activated by LPS in the presence of vehicle or indicated concentrations of BX-795. Supernatants were collected and applied to fresh MDM that were infected by ADA in the presence of vehicle or BX-795. N = 2. (C) MDM were activated by LPS in the presence of vehicle or BX-795 (LPS + BX-795 supt, grey bar). Supernatants were collected and applied to fresh MDM that were infected by ADA in the presence of vehicle or BX-795 (ADA+ BX-795, grey striped bar). N>3. All infected cells were harvested after 24 h for measurement of HIV-1 DNA, means and standard deviations are plotted. * p<0.05, ** p<0.005, *** p<0.0005 in Student's *t* test comparing virus burdens in the infected cell systems indicated.

The TBK1/IRF-3/interferon-β (IFN-β) signaling pathway is well documented for its critical roles in mediating TLR-induced antiviral responses [21], so we tested its involvement in the TLR induced anti-HIV-1 response described here. The antiviral response to LPS was reversed in MDM treated with LPS and the TBK1 inhibitor, BX-795 (Fig. 11A). To test the role of TBK1 in the LPS-induced secretion of antiviral factors, supernatants were collected from MDM treated with LPS, different doses of BX-795, or both and then used for treatment of MDM during ADA infection (Fig 11B). BX-795 highly significantly reduced the level of antiviral activity in LPS supernatants in dose response (p<0.005), although even at the highest dose of the BX-795, antiviral activity was detected. Note that BX-795 had no effect upon ADA replication, as shown in BX-795 dose response conducted in the presence of control MDM supernatant. To determine whether TBK1 is required for the response to LPS in contrast to the antiviral activity against HIV-1 we again separated these two phases of cellular activity. To test the production of antiviral factors, MDM were treated with vehicle or LPS in the presence or absence of BX-795 and their supernatants were harvested. Antiviral activity in supernatants was tested during ADA infection of MDM conducted in the presence or absence of BX-795 (Fig. 11C). BX-795 blocked the LPS induced production of antiviral factors by MDM. However when antiviral factors induced in MDM by LPS were tested during infection in the presence of BX-795, they largely maintained activity and inhibited HIV-1 replication, indicating that TBK1 is not essential for their antiviral function (Fig 11C).

**Figure 12:**
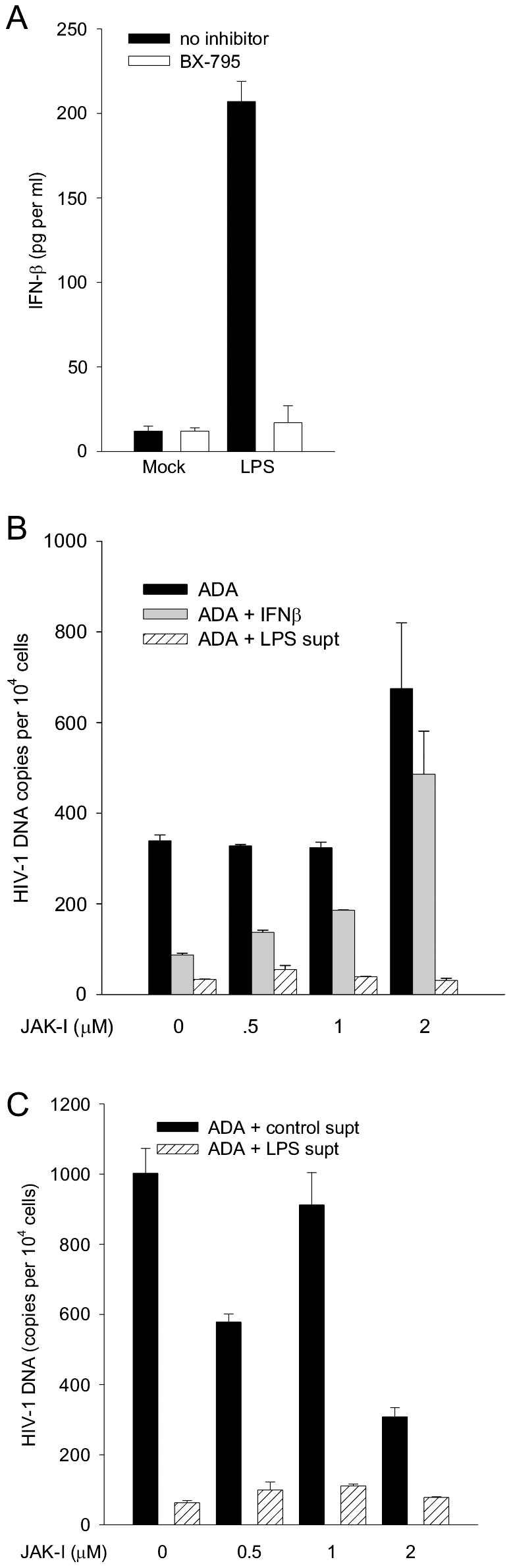
IFN-β is dispensable for the anti-HIV-1 response induced by LPS. (A) MDM were activated by LPS in the presence of vehicle or BX-795, supernatants were collected for the measurement of IFN-β. N = 2. (B) MDM were infected by ADA at the indicated concentrations of JAK-I in the presence of vehicle, supernatants of LPS-activated cells, or recombinant IFN-β. N = 2. (C) MDM were activated by LPS in the presence of vehicle or indicated concentrations of JAK-I and their supernatants were collected and applied to fresh MDM that were infected by ADA. N = 3. All infected cells were harvested after 24 h for measurement of HIV-1 DNA, means and standard deviations are plotted.

The requirement for TBK1 for LPS-induction of anti-HIV-1 factors is consistent with the possibility that IFN-β is responsible for some or all of the antiviral activity [21]. To evaluate this proposition, we first tested whether IFN-β was produced in response to LPS and whether its induction was sensitive to BX-795, IFN-β was measured by Elisa (Fig. 12A). We found that LPS induced 207 pg IFN-β per ml, using this assay equivalent to less than 100 inhibitory units (IU). Parallel Elisa failed to detect IFN-α with a limit of detection of 12 pg per ml. The induction of IFN-β was sensitive to inhibition by BX-795, consistent with a role for IFN-β in the antiviral state described here. Because IFN-β induces a large suite of antiviral activities through JAK/STAT signaling, we tested the requirement for JAK/STAT in mediation of anti-HIV-1 factor activity induced by LPS. We conducted a dose response of recombinant IFN-β in MDM and observed that 1000 IU were required for optimal STAT-1 phosphorylation (not shown). The supernatant from LPS-activated MDM was compared to 1000 IU recombinant IFN-β for the ability to inhibit ADA infection of MDM in the presence of graded doses of JAK inhibitor (JAK-I) (Fig. 12B). Both supernatant of LPS-activated MDM and recombinant IFN-β significantly inhibited HIV-1 infection prior to viral DNA synthesis (p<0.005), note that the effective dose of IFN-β was more than 10 times its concentration in the activated MDM supernatant. The antiviral activity of IFN-β was significantly reduced by the JAK-I in dose response, with no significant inhibition of HIV-1 by IFN-β observed in the presence of 5 µM JAK-I. In striking contrast, the antiviral activity produced by LPS-activated MDM maintained highly significant inhibition (p< 0.001-0.02) at all doses of the JAK-I tested. However, induction by LPS may involve JAK/STAT signaling to produce IFN-β or other antiviral factors. To test this possibility we investigated the sensitivity of MDM activation by LPS to JAK-I. MDM were activated by LPS in the presence of graded doses of JAK-I and their supernatants were collected. The supernatants were then tested for their antiviral activity (Fig. 12C). Inhibition of JAK/STAT signaling did not affect the extent of antiviral activity produced by LPS-activated MDM with significant inhibitory activity present in supernatants (p<0.005) produced at all doses of the JAK-I. These findings indicate that the TLR ligand-induced antiviral activity described here is different from IFN-β and from other factors requiring JAK/STAT signaling in their induction.

**Figure 13:**
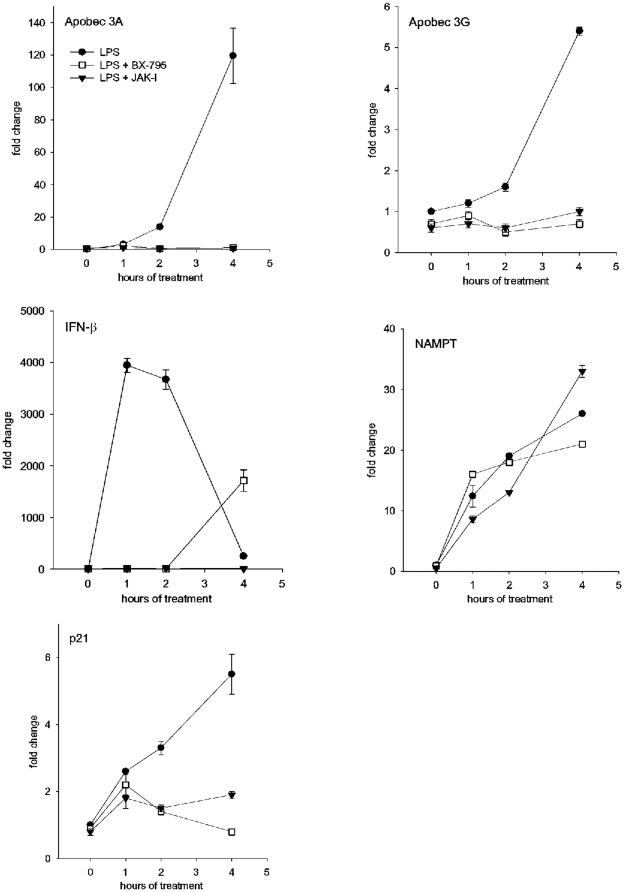
TBK1 and JAK/STAT are either both required or both dispensable for LPS-induction of previously described antiviral factors. MDM were activated by LPS in the presence of vehicle, BX-795, or JAK-I and cells were harvested at the indicated times for real-time PCR amplification of the specific transcripts indicated, standardized by GAPDH transcripts as described in Materials and Methods, means and standard deviations are plotted. N = 2.

Given the difference in their effects upon antiviral factor production, BX-795 and JAK-I provide a tool to identify the active factor. Expression of the TLR-linked antiviral factor should be induced in MDM by LPS in the presence of JAK-I, but its LPS-induction should be reduced by treatment of cells with BX-795. We investigated five defined anti-HIV-1 factors that can be expressed in macrophages [16], [22] for their induction by LPS and the sensitivity of this induction to inhibition by BX-795 or JAK-I; transcripts were measured over four hours induction using real-time PCR (Fig. 13). APOBEC 3A, APOBEC 3G, IFN-β, NAMPT, and p21^Cip1^ were each induced in MDM by LPS to varying degrees from 4000-fold for IFN-β to roughly 5-fold for APOBEC 3G. The expression of NAMPT was largely resistant to the signaling inhibitors, the expression of the four other transcripts was sensitive to both inhibitors. These results indicate that the antiviral activity studied here that requires TBK1 but is independent of JAK/STAT signaling is different from APOBEC 3A, APOBEC 3G, IFN-β, NAMPT, and p21^Cip1^ because of the signaling requirements for their expression following LPS activation.

## Discussion

We find that upon triggering any of three TLR, MDM mount an innate immune response that inhibits HIV-1 infection, they secrete factor(s) that induce a similar antiviral state in untreated MDM. Lymphocytes neither express nor respond to this antiviral activity. TLR-activated MDM permit HIV-1 entry but block virus replication prior to reverse transcription. The cell-type specificity, site of action, and requirement for signaling intermediates suggest that the antiviral activity observed is novel.

The robust response described here was observed in multiple cell donors, triggered by multiple TLR ligands, and active against multiple HIV-1 strains. Activated MDM restrict HIV-1 replication and they also secrete antiviral activity. Because the antiviral activity can be detected in supernatants of MDM within an hour of their exposure to TLR ligands, it is possible that an antiviral factor is secreted, internalizes in infected cells, and then arrests HIV-1 replication after virus entry. In contrast, PBL do not respond to TLR ligands by inhibition of HIV-1 infection and MDM-derived antiviral factors do not affect HIV-1 infection of PBL. This suggests that the antiviral factor described here is different from previously reported antiviral factors APOBEC 3G, β-chemokines, and SLPI that inhibit HIV-1 replication in PBL [22], [23].

Activated macrophages, including macrophages activated by IFN-β[24] or dsRNA [12], produce β-chemokines [25] that antagonize R5 HIV-1 binding to CCR5 and block infection at entry [26]. LPS-activated MDM also have been reported to directly down-regulate CCR5 expression and acquire resistance to R5 HIV-1 entry [6]. However we find that MDM activated by LPS allow efficient HIV-1 entry, placing the activity of the antiviral factor post-entry but prior to reverse transcription. p21^Cip1^ expression by macrophages has been found to have an antiviral effect similar to ours in some studies [27] but to enhance HIV-1 infection of macrophages in other studies [28]. The antiviral activity investigated here can be distinguished from p21^Cip1^ because the induction of p21^Cip1^by LPS is sensitive to inhibition by JAK-I. (Fig. 13).

To identify the route of induction of antiviral activity by MDM, we investigated the requirement for certain intermediates in TLR signaling. Induction of antiviral activity here requires TBK1 and can utilize the combined effects of p38 and JNK MAPK. In contrast, it was striking that both the NF-κB and the JAK/STAT pathways that are prominent in TLR response networks were dispensable for the anti-HIV-1 activity induced in MDM. On that basis, β-chemokines, some antiviral cytokines, and IFN-related genes that can affect HIV-1 replication [20], [27] are not likely to be responsible for the HIV-1 inhibition seen under our conditions. Similarly, we found that LPS-induction of expression of previously described antiviral factors APOBEC 3A, APOBEC 3G, IFN-β, p21^Cip1^ [16] is inconsistent with the anti-HIV-1 activity here because their expression requires both TBK-1 and JAK/STAT as noted above, while NAMPT [22] can be ruled out as the mediator observed here because its expression requires neither TBK-1 nor JAK/STAT. Taken together, our findings suggest that there is an unidentified factor produced in response to TLR ligands that can arrest HIV-1 infection of macrophages after virus entry and before reverse transcription.

Responses to TLR ligands have been described to underlie HIV-1 or SIV pathogenesis [11], [29], [30] or to provide some protection against HIV-1 replication [31]. The ligands examined here may be encountered during HIV-1 infection of human beings: LPS through microbial translocation from the gut [29], single stranded RNA in the viral genome, and dsRNA as an intermediate during reverse transcription. We speculate that the response mounted by MDM to TLR ligands may contribute to HIV-1 control, particularly in tissues where HIV-1 predominantly replicates in macrophages like the brain or the lung. Definition of the antiviral factor mediating protection may provide an additional approach to control or prevent HIV-1 spread.

## Materials and Methods

### Ethics statement

Healthy HIV-1 seronegative blood donors signed informed consent forms that were approved by the University of Nebraska Medical Center Institutional Review Board where blood was collected, cells separated by centrifugal elutriation, and then transferred for use in these studies for a fee. The studies reported here using human peripheral blood monocytes and lymphocytes were granted exempt status by the St. Luke's-Roosevelt Institutional Review Board under qualifications listed in section 45.101 (b) (4).

### Reagents

Lipopolysaccharide (LPS) from Escherichia coli 0127:B8 and PS-1145 were purchased from Sigma. Polyinosinic acid • polycytidylic acid, sodium salt (dsRNA), CAPE, and JAK inhibitor I were purchased from Calbiochem. R848 was purchased from Invivogen. BX795 was purchased from Fisher Scientific. The p38 MAPK inhibitor, SB203580, and JNK MAPK inhibitor were purchased from EMD Chemicals. Recombinant human IFN-β was purchased from R & D Systems. Unless otherwise described, the following doses were used: 100 ng/ml for LPS, 25 µg/ml for dsRNA, 5 µg/ml for R848, 20 µM for PS-1145, 25 µg/ml for CAPE, 20 µM for SB203580, 10 µM for JNK inhibitor, 5 µM for BX795, 2 µM for JAK inhibitor, and 1000 IU of IFN-β.

### Cell culture

Human peripheral blood monocytes and peripheral blood lymphocytes were isolated by centrifugal elutriation from blood of more than 20 HIV-1-negative, healthy donors. Monocytes were differentiated to macrophages by culture in DMEM (10^6^ cells/ml) containing 10% human serum and 10% conditioned supernatant of Giant Cell Tumor cells (TIB-223™, ATCC) for 6 days. Floating cells were then washed away and attached macrophages were cultured in DMEM with 10% fetal bovine serum (FBS) overnight before being used for experiments. Under these conditions, untreated MDM were 93% viable, cells treated with LPS or inhibitors were 90-93% viable with the exception of CAPE treated cells at 72% viable and BX795 at 85% viable as determined by assay of lactate dehydrogenase release using a kit purchased from Promega and conducted according to the manufacturer's instructions. PBL were cultured in RPMI medium with 10% FBS in presence of PHA (5 µg/ml) and IL-2 (1 ng/ml) for 2 days. Activated PBL were then washed and re-suspended in RPMI with 10% FBS and IL-2 (1 ng/ml) before being used for experiments.

### Preparation of HIV-1 stocks

ADA and B.aL were prepared by infecting MDM, NL4-3 was prepared by infecting CEM-SS cells obtained from the AIDS Research Reagent Repository. From day 5 to day 13 after infection, conditioned supernatant was collected every two days. Supernatant was then centrifuged for 20 minutes at 3900 rpm at 4°C in Eppendorf Centrifuge 5810R to remove dead cells and cell debris. To concentrate the virus, cleaned supernatant was centrifuged again for 2 hours at 14000 rpm at 4°C in Beckman Coulter centrifuge Avanti J-E. Virus pellet was re-suspended in PBS (1∶100 of original volume), snap-frozen, and stored at −80°C. Virus titer was determined by HIV-1 p24 concentration by Elisa. YU-2 was prepared by transfecting viral vectors pUC19-YU-2 into 293 T cells using calcium phosphate. For Yu-2/BLaM-Vpr virus, pCMV-BLaM-Vpr (kindly provided by Dr. W. Greene, Gladstone Institute of Virology and Immunology, UCSF) and pAdVAntage (Promega) vectors were co-transfected together with pUC19-YU-2. Conditioned supernatant was collected on day 2 and day 3 after transfection. Virus was then concentrated as described above. All virus stocks were screened for mycoplasma and found to be negative.

### HIV-1 infection

For viruses prepared by direct infection and transfection, the doses of infection used were 0.05 pg p24/cell and 0.2 pg p24/cell, respectively. Cells were cultured with indicated dose of virus at 37°C, 5% CO_2_ for 1 hour. Supernatant was removed and the cells were washed once with PBS and continued to be cultured in appropriate medium.

### MDM supernatant transfer

MDM were cultured in presence of TLR ligands at 37°C, 5% CO_2_ for 10 minutes. Cells were then washed with cold PBS three times and returned to culture in fresh DMEM with 10% FBS as indicated. In experiments where cells were treated with signaling inhibitors before TLR ligation, inhibitors were replaced after washing. Conditioned supernatant was then collected which contained signaling inhibitors. To assay antiviral activity in supernatants, culture medium of untreated cells was removed and replaced by the test supernatant and HIV-1, as indicated. Unless otherwise stated, cells were washed after infection and the same conditioned supernatant, was added back to cultures.

### ELISA

To determine extracellular HIV-1 p24 concentration, supernatant from infected cells was collected as indicated and tested by ELISA using a kit from PerkinElmer. To determine extracellular IFN-β concentration, supernatant was collected 4 hours after LPS stimulation and tested by ELISA using a kit obtained from Interferonsource.

### Real-time PCR analysis of HIV-1 DNA

DNA from infected cells was prepared using DNAzol reagent (Invitrogen) following manufacturer's instructions. Real time PCR to amplify HIV-1 *gag* was conducted in ABI 7500 Real Time PCR System using primers for gag 5′-TGGGACCACAGGCTACACTAGA-3′ and 5′-CAGCCAAAACTCTTGCTTTATGG-3′ purchased from Invitrogen and probe 5′-TGATGACAGCATGCCAGGGAGTGG-3′ purchased from Applied Biosystems (ABI). For quantitation of *gag* a standard curve was conducted using HIV-1 plasmid DNA. DNA input was standardized by amplification of human β−globin in parallel using primer and probe set was from ABI: Hs00758889_s1.

Fluorescence Resonance Energy Transfer-based HIV-1 fusion assay (FRET assay) MDM were cultured in 12-well plates (10^6^ cells/well) in 1 ml of DMEM with 10% FBS. After being treated with LPS or TAK-779, cells were cultured with YU-2-BLaM-vpr virus (0.2 pg p24/cell) at 37°C, 5% of CO2 for 2 hours. Supernatant was then removed and cells were washed twice with PBS and once with CO2-independent medium. 6× CCF2-AM solution was prepared using BLaM Loading Solutions with CCF2-AM substrate (K1085, from Invitrogen) following manufacturer's instructions. 40 µl of 6×CCF2-AM solution was then added into each well of cell culture (in 200 µl of CO_2_-independent medium). Cells were then incubated at room temperature in the dark for 1 hour, followed by washing once with development medium (2.5 mM of probenecid and 10% FBS in CO_2_ -independent medium), and continued to be cultured in development medium (1 ml/well) at room temperature in the dark for 16 hours. Development was stopped by removing development medium and washing cells with PBS. Cells were then collected by cell scraper (Fisher Scientific) and re-suspended thoroughly in PBS followed by fixation with 1.2% of paraformaldehyde for 2 hours at 4°C. Fusion of virion was analyzed by flow cytometry detection of fluorescence emission at 520 nm and 447 nm with BD LSRII Flow Cytometer. Data were analyzed with Flowjo Software.

### Real-time PCR analysis of mRNA expression

RNA from treated cells was prepared using TRIzol reagent (Invitrogen) and then purified using RNeasy Mini Kit (Qiagen) following manufacturer's instructions. Revere transcription was then carried using SuperScriptTM First-Strand Synthesis System for RT-PCR (Invitrogen). cDNA was then subjected to real-time PCR analysis of mRNA expression by a ΔCt method with ABI 7500 Real Time PCR System. For IFN-β, primers were synthesized from Invitrogen: 5′-GCCAGGAGGTTCTCAACAAT-3′ and 5′-CTTTGCTATTTTCAGACAAGATTCA-3′; and probe was from Roche Universal Probe Library No. 20. For the other genes, primer and probe set for TaqMan Gene Expression Assays from ABI were used: cyclin-dependent kinase inhibitor 1A (p21, Hs00355782_m1), apolipoprotein B mRNA editing enzyme, catalytic polypeptide-like 3A (APOBEC 3A, Hs00377444_m1), apolipoprotein B mRNA editing enzyme, catalytic polypeptide-like 3G (APOBEC 3G, Hs00222415_m1), and nicotinamide phosphoribosyl-transferase (NAMPT, Hs00237184_m1). GAPDH (ABI primer and probe set, 4352934E) was used as an endogenous standard.

### Replicas and statistics

Every experiment was performed independently in cells from two or more donors. The number of donors employed is noted in each figure legend as N. As indicated, the differences between virus DNA burdens in different systems were tested by Students *t* test.
